# Alterations in bile acids as metabolic signatures in the patients with human adenovirus type 7 infection

**DOI:** 10.3389/fmed.2022.896409

**Published:** 2022-09-07

**Authors:** Wen Xu, Juan Du, Ting-Ting Wei, Lin-Yi Chen, Xin-Xin Yang, Tu Bo, Han-Yu Liu, Ming-Zhu Xie, Tian-Shuo Zhao, Jun-Lian Yang, Fuqiang Cui, Wei-Wei Chen, Qing-Bin Lu

**Affiliations:** ^1^Department of Infectious Disease, The Fifth Medical Center of Chinese People’s Liberation Army General Hospital, National Clinical Research Center for Infectious Diseases, Beijing, China; ^2^Department of Laboratorial Science and Technology and Vaccine Research Center, School of Public Health, Peking University, Beijing, China; ^3^Global Center for Infectious Disease and Policy Research, Peking University, Beijing, China

**Keywords:** human adenovirus, metabolomics, bile acids, cytokines, acute respiratory tract infection

## Abstract

**Objectives:**

The changes in metabolism by human adenovirus (HAdV) infection was unclear. The potential mechanism of HAdV-7 causing acute respiratory tract infection was explored.

**Methods:**

Totally 35 patients with HAdV-7 infection, 32 asymptomatic cases with HAdV-7 and 14 healthy controls were enrolled from an outbreak of HAdV-7 in the army. The serum samples were analyzed by untargeted and targeted metabolomics. The effects of differential metabolites were verified on HAdV-7 replication in an A549 cell line.

**Results:**

The untargeted metabolomics analysis revealed more significant changes in the classes of sphingolipids, polyketides, glycerolipids, fatty acyls, and carboxylic acids and their derivatives in the patients with HAdV-7 than in healthy controls. Two key metabolic pathways of secondary and primary bile acid biosynthesis were noted from pathway enrichment analysis. Targeted metabolomics analysis showed that the levels of unconjugated bile acids in the patients were significantly lower, while the levels of glyco- and tauro- conjugated bile acids in patients and asymptomatic cases were higher than those in the healthy controls. The profiles of cytokines and peripheral lymphocyte subsets obviously varied at different levels of bile acids, with significant differences after HAdV-7 infection. A cell verification test demonstrated that the replication of HAdV-7 significantly reduced when GCDCA and TCA were added.

**Conclusion:**

Bile acids inhibited HAdV-7 replication *in vitro*. Alterations in bile acids was metabolic signatures of HAdV-7 infected subjects, and our results suggested bile acids might play protective roles against HAdV-7 infection.

## Introduction

Human adenovirus (HAdV) is an important pathogen causing acute respiratory tract infection, which can also affect the digestive tract, urinary tract, eye and myocardium (1). Nearly 20% of HAdV infections are caused by HAdV-7, of which outbreaks mainly at kindergarten classroom, in the community and at military training bases (2–4). Reemergence of HAdV-7 was reported recently and led to fatal pneumonia cases in China, which has also been reported in the US (5–8). HAdV-7d, especially HAdV-7d2 emerged in China and caused higher severe and mortality rates (8, 9). To date, no approved antiviral therapy is available for HAdV infections. An in-depth understanding of HAdV infection therefore is needed to develop more therapeutic options to combat HAdV infection.

Metabolomic analysis has been shown to be a powerful tool for the diagnosis, treatment, and prevention of human diseases (10, 11), as well as an effective tool for biomarker screening, and characterization of biological pathways (12). Studies have confirmed that HAdV infections increased glucose uptake and lactic acid production (13, 14). Increases in acetyl-CoA, glutamine catabolism and the tricarboxylic acid cycle have also been reported. Another study showed that transcripts of the glutamine transporter genes ASCT2 and LAT1 increased in HAdV-5-infected cells (15). A variety of metabolism related proteins with differential expressions throughout infection was identified, such as upregulated levels of serine glycine biosynthesis and mannose metabolism (16). However, most studies have focused on the cell lines, not on humans.

This study revealed the serum metabolite profiles of the patients with HAdV-7 infection to explore the potential mechanism causing acute respiratory tract infection and establish a diagnostic model from these metabolic biomarkers to distinguish patients from the healthy controls as early as possible, which would be helpful for the control and therapy of HAdV infection.

## Materials and methods

### Study subjects

Patients with HAdV-7 infection in a military camp with a HAdV outbreak were enrolled in this study, including the patients and asymptomatic cases. Patients with acute respiratory tract infection were positive for HAdV-7 and presented with influenza-like symptoms or pneumonia. Asymptomatic patients were positive for HAdV-7 but without any symptoms. Healthy controls who came from other troops and were negative for HAdV infection were enrolled as controls. Informed consent was obtained from all the participants, and the study protocol was approved by the Human Ethics Committee of Peking University Institutional Review Board Office (IRB00001052-19005) and the Ethics Committee of Chinese People’s Liberation Army 302 Hospital (2018032D).

### Information and samples of the study subjects

Demographic, clinical, and laboratory data were collected by reviewing of clinical records, nursing charts, and laboratory tests of the patients. Throat swabs and peripheral blood were collected from the subjects at approximately 6:00 before breakfast. The samples were processed within approximately 2 h and stored at −80°C. Blood samples were collected from patients at admission, at discharge from the hospital and after 1 month, while samples from asymptomatic cases were drawn in the early days of this outbreak and after 1 month. After the centrifugation (3,000 rpm) for 10 min at 4°C, 1.5 mL of each supernatant was transferred to fresh Eppendorf tubes and stored at −80°C until use.

### Detection of human adenovirus infection

Total nucleic acids were extracted from throat swabs using a viral DNA extraction kit (Cat No.: ER201-01, Transgen, China). HAdV was detected by a commercial HAdV detection kit (Cat No.: BP0211-32, Medical System, China) using an ABI 7500 PCR system. A combination of probe and primers were used for the detection of HAdV in patients. The probe (5′- ATCAACCACCTGCCTGCTCATA –3′) was modified by fluorophore FAM at the 5′ end and quencher BHQ1 at the 3′ end. The length of product amplified by the forward primer (5′- CTGAGGGATACAAGGATC –3′) and reverse primer (5′- GGCTTTGTAGTCAGTGTA –3′) was 98 base pairs. The samples with HAdV-7 infection were confirmed by the sequencing of Hexon gene, Fiber gene, Penton gene, or whole genome. The amplification primers of Hexon gene were HVR-F (5′-CAGGATGCTTCGGAGTACCTGAG-3′) and HVR-R (5′-TTTCTGAAGTTCCACTCGTAGGTGTA-3′). Primers Fiber-F (5′-CCCTCTTCCCAACTCTGGTA-3′) and Fiber-R (5′-GGGGAGGCAAAATAACTACTCG-3′) were amplified for Fiber gene. For Penton gen, the primer of Penton-F (5′-CTATCAGAACGACCACAGCAACTT-3′) and Penton-R (5′-TCCCGTGATCTGTGAGAGCRG-3′) was used in this study.

### Determination of lymphocyte subset counts

The absolute counts of lymphocyte subsets in the peripheral blood of patients were determined by a MultiTest IMK kit (Cat No.: 662965; BD, San Diego, CA, United States) with a trucount tube (BD, San Diego, CA, United States) according to the manufacturer’s instructions.

### Peripheral blood mononuclear cell isolation

Peripheral blood mononuclear cells (PBMCs) were isolated by gradient centrifugation using a Ficoll-Paque PLUS kit (GE Healthcare Life Sciences, Pittsburgh, PA, United States). Briefly, the blood was diluted with the same volume of phosphate buffered saline (PBS) (Solarbio, Beijing, China). The diluted samples were carefully layered onto the Ficoll-Paque medium solution, and then centrifuged at 400 × g for 35 min at 20°C. Afterward, the mononuclear cell layers were transferred into new tubes and washed with PBS twice. The brake of the centrifuge was turned off to minimize disturb of the mononuclear layer.

### Sample preparation for LC-MS analysis

All the samples were thawed at 4°C, and quality control (QC) samples were prepared by pooling aliquots (10 μL) of each sample. Acetonitrile (800 μL) was added to each serum (200 μL) and vortexed for 1 min. The mixtures were incubated at room temperature for 1 min and then centrifuged at 14,000 rpm for 10 min at 4°C. The resulting clear supernatants were transferred to UPLC vials, and then stored at 4°C before detection. The pretreatment of the QC samples was the same as that of the real samples.

### LC-MS analysis of the samples

Reversed-phase analysis was performed on a Waters ACQUITY Ultra Performance LC system using an ACQUITY UPLC BEH C18 analytical column (detailed in Supplementary materials). The QC samples were injected at regular intervals (every 10 samples) throughout the analytical run.

### Virus isolation and bile acid stimulation

A549 cells were thawed and cultured according to methods on the ATCC website. Briefly, the cells were cultured in DMEM medium supplemented with 10% fetal bovine serum, 100 U/mL penicillin and 100 μg/mL streptomycin. After reaching confluence, throat swab samples were collected from the patients. Viruses were harvested when significant cytotoxicity was observed and the TCID50 was determined by the Reed-Muench method. The genome of the isolated virus was sequenced and identified as HAdV-7d using the BLAST program of the NCBI website.

All bile acids were purchased from Sigma Aldrich. Taurocholic acid (TCA) and glycochenodeoxycholic acid (GCDCA) were dissolved in cell medium. A549 cells were stimulated with HAdV and bile acids simultaneously for 24 h. Then, the cells and supernatants were harvested. For quantification of HAdV in the samples, HAdV copies of standards were first determined using droplets of digital PCR mixes (TargetingOne, Beijing, China) and a TargetingOne digital PCR detection system (TargetingOne, Beijing, China). Then, all the samples and standards were tested *via* real-time PCR. A standard curve was generated, and the copy numbers of the samples were determined. The total copy number of HAdV was calculated as the sum of the copy numbers in the cell lysates and supernatants.

The cytotoxic effects of bile acids at the concentrations used in our study were evaluated by a Cell Counting Kit-8 (CCK-8, Dojindo, Japan) and microscopy observations. A CCK-8 assay was performed according to the instructions provided by the manufacturer.

### Detection of serum cytokines in patients and asymptomatic cases

Serum concentrations of interleukin (IL) 6 (Abcam, United States, ab46027), interferon (IFN) γ (Abcam, United States, ab46048), interferon-inducible protein (IP) 10 (Abcam, United States, ab173194), interferon α 1 (Abcam, United States, ab213479), IL-10 (Abcamn United States, ab100549), and HMGB-1 (IBL, Germany, ST51011), and the serum receptor for advanced glycation end-product (sRAGE, R&D, United States, DRG00) were detected *via* commercial ELISA kits following the instructions provided by the manufacturers (detailed in Supplementary materials).

### Data processing and statistical analysis

Descriptive statistics were calculated for all the variables; the continuous variables were described as the means and standard deviations (SDs) with a normal distribution or as medians and interquartile ranges (IQRs) with an abnormal distribution, and the categorical variables were described as frequencies and proportions.

The metabolomics data were analyzed by OPLS-DA and PCA models. Potential biomarkers for differentiating each group from the control group were selected according to the variable importance in the projection (VIP) values. Comparisons of the variables among the three groups were performed by one-way analysis of variance or the Kruskal-Wallis test. All analyses were performed using STATA 17.0 (Stata Corp. LP, College Station, TX, United States). Column scatter plots were generated using GraphPad Prism 8.0, and the scatter plots of correlations and box plots were generated using Microsoft Excel 2019. A two-sided *P* < 0.05 was considered to indicate statistical significance.

## Results

### Dramatic and clinical manifestations

A total of 35 patients with HAdV-7 infection, 32 asymptomatic cases with HAdV-7 and 14 healthy controls were enrolled in the study (Supplementary Table 1). All the cases were male, and the means of age and BMI were comparable among the three groups. No significant differences were observed in the frequencies of smoking or drinking. Among the 35 patients, 21 were diagnosed with upper respiratory tract infection, and 14 were diagnosed with pneumonia. The top five symptoms and signs were pharyngalgia (91.4%), fever (85.7%), cough (80.0%), diarrhea (45.7%) and expectoration (42.9%).

### Untargeted metabolomics

**FIGURE 1:**
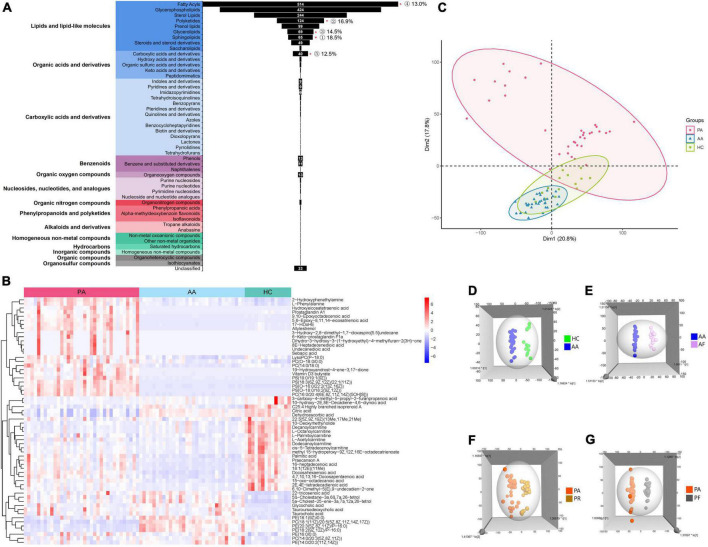
Analysis of untargeted metabolomics between cases and healthy controls. **(A)** Shows the classification of all metabolites that were screened. “*”*p* < 0.05; the number of % represent for the ratio of the number of differential metabolites with *P* < 0.05 to each class. **(B)** Shows the clustering results of the metabolites at the top 60 of VIP among PA, AA, and HC. **(C)** Shows the PCA of samples from the three groups. **(D)** Shows the 3D OPLS-DA scatter plot for HC and AA. **(E)** Shows the 3D OPLS-DA scatter plot between AA and AF. **(F)** Shows the 3D OPLS-DA scatter plot between the PA and PR. **(G)** Shows the 3D OPLS-DA scatter plot between PA and PF. PA, patients in the acute stage; AA, asymptomatic cases at the acute stage; HC, healthy controls; AF, asymptomatic cases at the follow-up stage; PR, patients at the recovery stage; PF, patients in the follow-up stage.

All the 169 samples from the three groups were subjected to the untargeted metabolomics (Supplementary Figure 1). The good stability and repeatability of the profiled information was demonstrated over time by quality control samples (Supplementary Figure 2). The metabolites in these groups varied greatly, and 1,781 metabolites were screened after a series of pretreatments and subsequently classified into 14 super classes and 49 classes (Figure 1A). The top five detected classes were fatty acyls, glycerophospholipids, sterol lipids, polyketides, and prenol lipids. Compared to healthy controls, the HAdV-7 patients had more significant changes in the classes of sphingolipids, polyketides, glycerolipids, fatty acyls, and carboxylic acids and their derivatives (*P* < 0.05). A heatmap of all the metabolites in the three groups is shown in Supplementary Figure 3. To give a clear picture, the top 60 metabolites were filtered with a variable importance in projection (VIP) value of > 1 or a *P*-value < 0.05 in comparisons among the three groups at the acute stage (Figure 1B). Both of hierarchical clustering plots showed different metabolic signatures among the three groups.

We performed the unsupervised PCA and constructed supervised OPLS-DA models to analyze the serum metabolic profiles among the three groups (Figures 1C–G). The PCA plot showed that the patient group separated from the other two groups, and the asymptomatic cases were relatively close to the healthy controls with overlapping points (Figure 1C). However, the asymptomatic cases were distinct from the healthy controls according to the OPLS-DA models (Figure 1D). Obvious changes in the serum metabolism were observed in the two groups at the acute stage, the recovery stage and the follow-up stage (Figures 1E–G).

**FIGURE 2:**
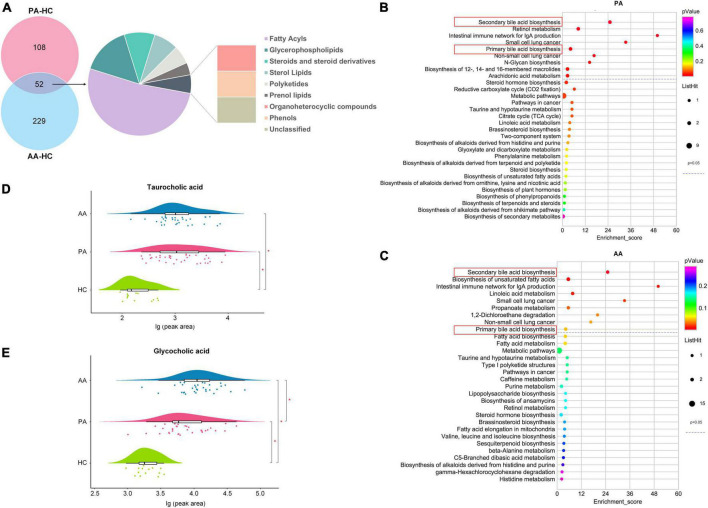
Differential metabolites and metabolic pathway enrichment *via* untargeted metabolomics analysis. **(A)** Shows the classes of differentially accumulated metabolites between PA and AA and HC. **(B,C)** Show the concentrations of the taurocholic acid and glycocholic acid among PA, AA, and HC, respectively. **(D,E)** Show the enrichment of PA and AA metabolic pathways, respectively. PA, patients in the acute stage; AA, asymptomatic cases in the acute stage; HC, healthy controls.

According to a VIP > 1 by the OPLS-DA model and a *P*-value < 0.05 obtained by FDR correction after single-factor analysis, 160 and 281 metabolites were screened between the patients and healthy controls and between the asymptomatic cases and healthy controls, respectively (Figure 2A). A total of 52 metabolites were detected between the two comparison groups, which spanned nine classes, mainly fatty acyls (27 metabolites), glycerophospholipids (8 metabolites) and steroids and steroid derivatives (5 metabolites). All the potential metabolites were identified and queried through the KEGG databases,^1^ and significantly enriched metabolic pathways were determined with nine for the patients and nine for asymptomatic cases compared to healthy controls (Figures 2B,C). Two key metabolic pathways of secondary and primary bile acid biosynthesis involving TCA and glycocholic acid (GCA), were noted between the results of the two pathway enrichment analyses. Compared to those in the healthy controls, the relative concentrations of TCA and GCA in the two groups were higher at the acute stage (Figures 2D,E).

### Targeted metabolomics

**FIGURE 3:**
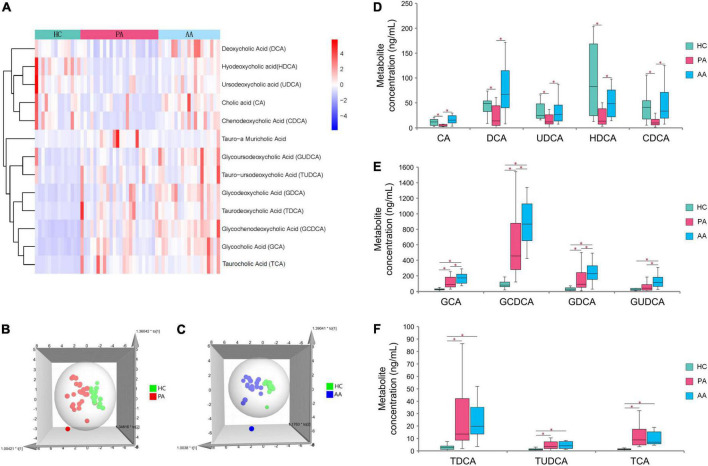
Analysis of targeted metabolomics between cases and healthy controls. **(A)** Shows the clustering results of the bile acid P among PA, AA, and HC. **(B,C)** Show the 3D OPLS-DA scatter plot for PA, AA, and HC, respectively. **(D–F)** Show the concentrations of thirteen bile acids in PA, AA, and HC. PA: patients in the acute stage. AA, asymptomatic cases at the acute stage; HC, healthy controls.

Untargeted metabolomics analysis revealed that bile acids, especially including TCA and GCA, played an important role in HAdV-7 infection. Therefore, we performed absolute quantification of thirteen targeted bile acids in some of the subjects in the study (Supplementary Figure 1). The resulting hierarchical clustering plots of bile acids at the acute stage showed obvious clustering features (Figure 3A). There was a certain separation trend of bile acids by the OPLS-DA model between the healthy controls and the other two groups, respectively (Figures 3B,C). According to the concentrations of bile acids at the acute stage among the three groups, the change pattern of bile acids was divided into three different types (Figures 3D–F). Five unconjugated bile acids in the patients, including cholic acid (CA), deoxycholic acid (DCA), ursodeoxycholic acid (UDCA), hyodeoxycholic acid (HDCA), and chenodeoxycholic acid (CDCA), were significantly lower than those in the asymptomatic cases or healthy controls. For conjugated bile acids, the concentrations of glycodeoxycholic acid (GCA), GCDCA, glycodeoxycholic acid (GDCA) and glycoursodeoxycholic acid (GUDCA) in the asymptomatic cases were significantly higher than those in patients and healthy controls. The concentrations of three bile acids of taurodeoxycholic acid (TDCA), tauroursodeoxycholic acid (TUDCA) and TCA, in the patients and asymptomatic cases were significantly higher than those in the healthy controls with significant differences.

### Cytokines, peripheral lymphocyte subsets and bile acids

**FIGURE 4:**
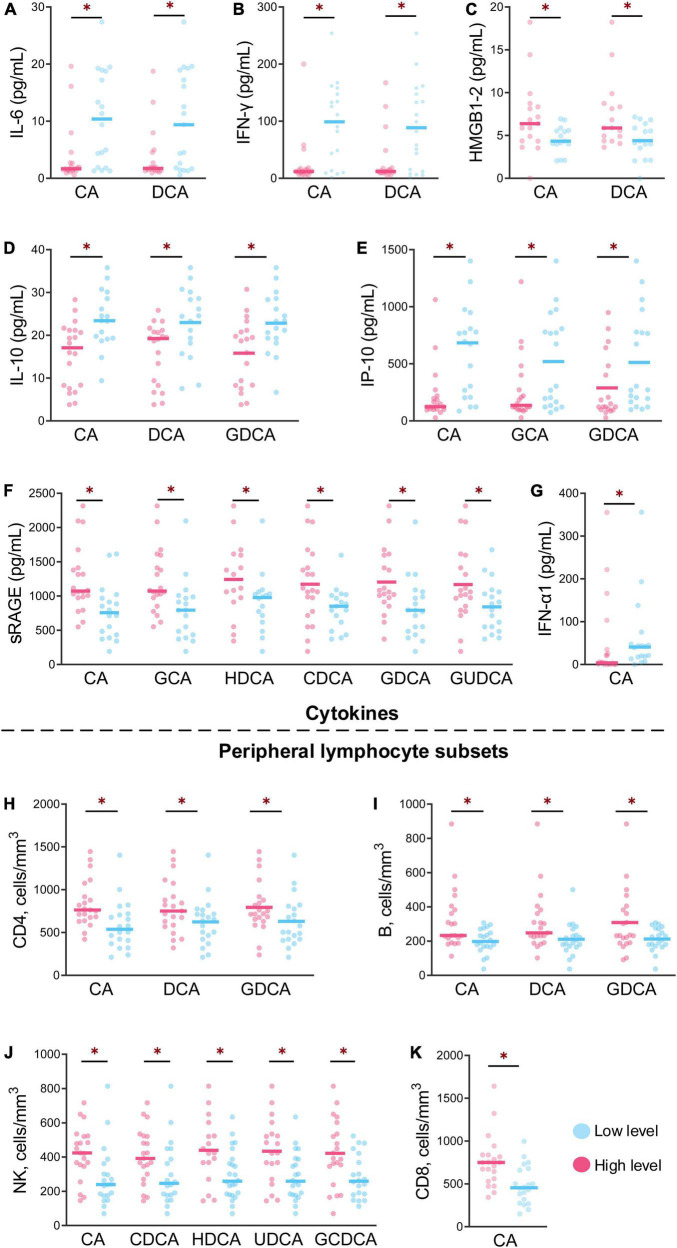
The levels of cytokines and peripheral lymphocyte subsets at the different levels of bile acids were significantly different between the patients and asymptomatic cases. **(A–G)** Show the levels of seven cytokines between the high and low of bile acid levels with significant differences. **(H–K)** Show the contents of four peripheral lymphocyte subsets between high and low bile acid levels with significant difference *p < 0.05.

The profiles of cytokines and peripheral lymphocyte subsets obviously varied in the different groups from different levels of bile acids, with significant differences after HAdV-7 infection (Figure 4). For CA and DCA, the levels of IL-6 and IFN-γ were lower in the high-level group than in the low-level group, while the levels of HMGB1 and RAGE were higher in the high-level group (all *P* < 0.05, Figures 4A–C). The levels of IL-10 and IP-10 in the high-level group of CA, DCA and GDCA were lower than those in the low-level group (Figures 4D,E), but the counts of CD4^+^ and B cells exhibited the opposite trend (Figures 4H,I). The level of RAGE and the count of NK cells were higher in the high-level group of six bile acids and five bile acids, respectively (Figures 4F,J).

The level of IFN-α1 was higher in the low-level CA group, but the CD8^+^ T cells count levels exhibited the opposite trend (Figures 4G,K).

### Glycochenodeoxycholic acid and taurocholic acid inhibit human adenovirus-7 replication

**FIGURE 5:**
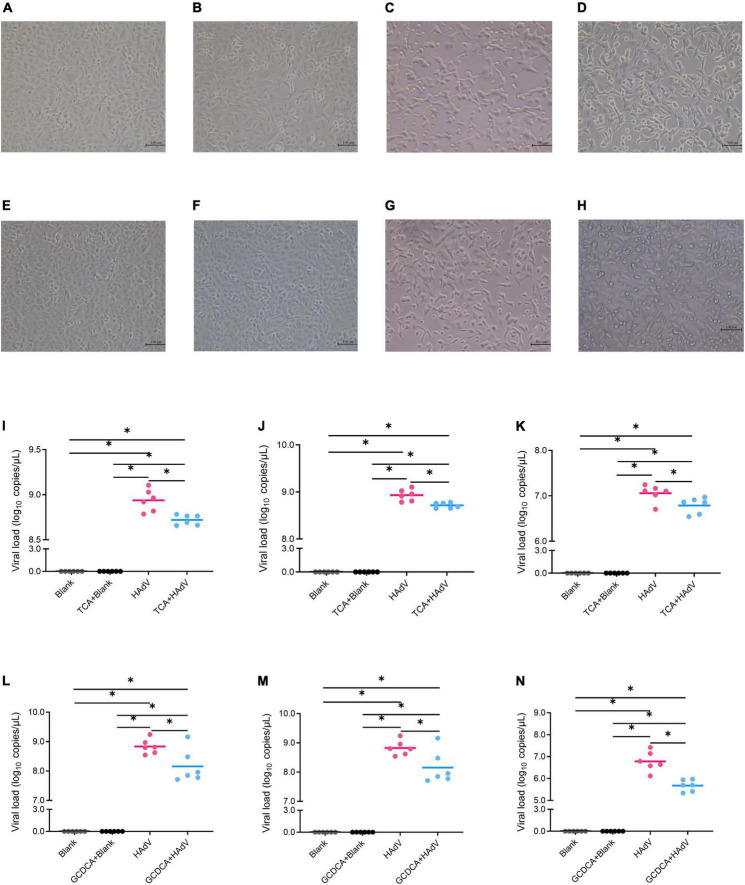
Effects of bile acids, including TCA and GCDCA, on HAdV-7 replication in the A549 cell line. **(A–H)** Show the cytopathic effect *via* microscopy observations; **(A)** the blank control for TCA test; **(B)** the blank control with TCA; **(C)** the HAdV-7-treated cells; **(D)** the HAdV-7-treated cell with TCA; **(E)** the blank control for GCDCA test; **(F)** the blank control with GCDCA; **(G)** the HAdV-7-treated cells; **(H)** the HAdV-7-treated cell with GCDCA). **(I–N)** Show the viral load in the blank control and HAdV-7 infection groups with or without bile acids at a MOI = 1. **(I)** Total viral loads with TCA; **(J)** viral loads with TCA in cell lysates; **(K)** viral loads with TCA in supernatants; **(L)** total viral loads with GCDCA; **(M)** viral loads with GCDCA in cell lysates; **(N)** viral loads with GCDCA in supernatants. All the experiments were repeated for six times *p < 0.05.

To investigate the effect of bile acids on HAdV-7 replication, we evaluated the effects of bile acids, including GCDCA and TCA, on HAdV-7 replication in an A549 cell line (Figure 5). A549 cells were cultured and infected with HAdV-7 at a MOI = 1. Compared with the blank control group, the virus infection group showed an obvious cytopathic effect, in which many cells fell off, aggregated and died. No significant cytotoxic effects of GCDCA (750 μM) or TCA (500 μM) were observed in our study (Figures 5B,F). Twenty-four hours post infection, lower viral loads were detected in the HAdV plus bile acids group than in the virus infection group, and the addition of GCDCA and TCA significantly reduced viral replications within the supernatant and cell lysate (Figures 5I–N).

## Discussion

Metabolic reprogramming of the host is an important aspect of many viral infections, but this process is worth further investigation to understand the underlying pathophysiology. HAdV infection-induced changes in cellular metabolism have been investigated *via* proteomics or transcriptomics (17). Furthermore, how HAdV infection alters the metabolism of patients remains to be elucidated.

Viral replication is associated with a high anabolic demand, which significantly alters the metabolism of target cells. By regulating lipid metabolism, such as changing phospholipids and sphingomyelins to promote the replication of hepatitis C virus, the viruses increasingly facilitate the entry, replication, assembly or secretion increasingly (18). Lipids are also important for host energy transfer, signal transduction, cell growth, apoptosis, inflammatory responses, etc. (19). Therefore, we hypothesized that the lipids and lipid-like molecules play the roles in HAdV-7 replication. By the untargeted metabolomics analysis, we found some significantly different metabolites between the HAdV-7 patients and the healthy controls, mainly lipids and lipid-like molecules, including sphingolipids, polyketides, glycerolipids, fatty acyls, carboxylic acids and their derivatives. Sphingolipids, which are present in all animals and plants, as well as in viruses, play vital roles in the regulation of membrane properties and are involved in many cellular functions, such as cell death, cell proliferation, apoptosis, inflammatory responses, and coagulation processes (20). Studies have shown that sphingolipids influence pulmonary leak and lung injury (21). As important components of the lipid bilayer, glycerolipids and glycerophospholipids not only maintain membrane stability, but also serve as secondary messengers to transduce cell signaling and then regulate immune activation and inflammation (19). These properties make these types of lipids important mediators in severe respiratory infection diseases, and an imbalanced lipid composition leads to the exacerbation of respiratory disease. HAdV infection usually causes mild symptoms, but the uncontrolled host inflammatory responses can lead to severe pneumonia or even death. Our results suggested that disruptions to lipid metabolism might be involved in the pathophysiology of HAdV infection although the underlying mechanisms were not clear. Lipids may be potential intervention targets for the treatment of HAdV infection.

We discovered that two key metabolic pathways of secondary and primary bile acid biosynthesis were associated with HAdV-7 infection. Bile acid metabolic pathways have been linked to many different types of viral infections. For examples, the levels of bile acids were shown to be higher in dengue fever patients than in healthy controls at the febrile and critical phases of infection (22). Further study showed that dengue hemorrhagic fever patients had even higher levels of bile acids than did dengue fever patients at both the critical and recovery phases of infection (23). In the HIV plasma metabolome, bile acids were higher than those in control subjects, acting as ligands of nuclear receptors that regulate metabolism and inflammation (24). Serum bile acids may represent a non-invasive marker of liver disease in patients with HCV (25). Interestingly, in this study, the targeted bile acids metabolomics analysis further revelated differential profiles of the unconjugated, glycine conjugated and taurine conjugated bile acids among healthy controls, patients and asymptomatic cases. The higher conjugated bile acid levels in HAdV-7 infection seemed that HAdV-7 infection profoundly triggered bile acid conjugation. However, the synthesis of unconjugated bile acids in HAdV-7 patients was relatively inadequate with the lower levels of the unconjugated bile acids in the patient. These results help us to understand the potential therapy method for HAdV-7d2, which causes a great burden on the severe pneumonia. Our results encouraged us to explore the role of bile acids in HAdV-7 infection.

Immune and inflammatory responses were shown to be associated with the outcome of respiratory infection, such as HAdV infection (26). The uncontrolled inflammatory responses lead to tissue damage and severe diseases. Bile acids are capable of inhibiting proinflammatory cytokine secretion and reducing tissue damage (27). *Via* the TGR5-cAMP-PKA pathway, bile acids suppress NLRP3 activation and subsequent inflammatory effects (28). In our study, the HAdV-7 infected patients had higher levels of inflammatory cytokines (IL-6, IFN-γ, IFN-α1, IP10, and IL-10) and a lower level of sRAGE, which were associated with reduced bile acid concentrations. RAGE is a multiligand receptor that binds to various danger-associated molecule patterns (DAMPs), and then mediates inflammatory responses in a range of sterile and non-sterile inflammatory diseases. sRAGE is a soluble form of RAGE and suppresses inflammatory signaling of RAGE through binding to RAGE ligands. Considering that HAdV-7 infected patients had lower levels of unconjugated and glycine-conjugated bile acids, our results suggested that higher bile acid levels mitigated excessive inflammatory responses and promoted disease recovery. As such, further evaluation of the effects of bile acid supplementation in HAdV-7 infection disease models or clinical trials is warranted.

Apart from the anti-inflammatory effect, our results showed that high bile acid levels were associated with high lymphocyte (CD4^+^ T cells, CD8^+^ T cells, B cells and NK cells) counts. Hu et al. reported that viral infection induced intracellular accumulation of bile acids in many different cell types and the bile acids promoted antiviral responses of target cells (29). Bile acids can regulate the immune functions of T cells and NK cells (30) and determine the differentiation of T cells (29). Our results suggested that higher bile acid levels promoted cellular immune responses against HAdV-7, but the underlying mechanism needs to be further investigated.

Luo et al. demonstrated that CDCA significantly reduced replication and inhibited activities against influenza A virus (31). A study revealed that CDCA markedly decreased rotavirus replication both *in vitro* and *in vivo* (32). Moreover, CA has been found to inhibit the replication, proliferation, and infection of Coxsackievirus-B3 (33). It has been discovered that the conjugated bile acids of TCDCA and GCDCA exhibit antiviral activity against cytomegalovirus replication (34). Then, a cell verification test demonstrated that the replication of HAdV-7 significantly reduced when GCDCA and TCA were added. Of course, the bile acids can also inhibit the HAdV infection of A549 cells resulting to the reduction of viral replication, which should be further confirmed. However, the effects of other bile acids were not confirmed in our study and dose-effect curves of GCDCA and TCA on the viral loads of HAdV were not performed, which needs to be further studied.

In summary, our results showed that HAdV-7 infection significantly altered host metabolism which provided more evidence for understanding the pathological mechanisms underlying HAdV-7 infection. Bile acid metabolic pathways were linked to inflammatory and immune response states in HAdV-7 infection. Bile acids also inhibited HAdV-7 replication *in vitro*. Our results suggested protective roles of some types of bile acids during HAdV-7 infection and further research is needed to elucidate how HAdV-7 infection regulates the bile acid metabolic pathway and to confirm the therapeutic effect of bile acids on HAdV-7 infection.

## Data availability statement

The original contributions presented in this study are publicly available. This data can be found here: metabolights; accession MTBLS4530 (private accession).

## Ethics statement

The studies involving human participants were reviewed and approved by the Human Ethics Committee of Peking University Institutional Review Board Office (IRB00001052-19005) the Ethics Committee of Chinese People’s Liberation Army 302 Hospital (2018032D). The patients/participants provided their written informed consent to participate in this study.

## Author contributions

Q-BL, W-WC, and FC: conceptualization and supervision. WX, JD, X-XY, H-YL, M-ZX, T-SZ, TB, J-LY, FC, W-WC, and Q-BL: methodology. WX, JD, L-YC, X-XY, TB, M-ZX, J-LY, and W-WC: investigation. WX, JD, H-YL, M-ZX, T-TW, T-SZ, FC, W-WC, and Q-BL: visualization. Q-BL, W-WC, and WX: funding acquisition. Q-BL, WX, W-WC, FC, and JD: project administration. WX, JD, L-YC, T-TW, FC, W-WC, and Q-BL: writing—original draft. Q-BL, W-WC, FC, WX, JD, L-YC, X-XY, TB, H-YL, M-ZX, T-TW, T-SZ, and J-LY: writing—review and editing. All authors contributed to the article and approved the submitted version.

## Footnotes

1
www.genome.jp/kegg/ligand.html
