# Supplementary material for: Alterations in bile acids as metabolic signatures in the patients with human adenovirus type 7 infection
Source: Front Med (Lausanne). 2022 Sep 7;9:896409. doi: 10.3389/fmed.2022.896409 (PMC9489940; doi:10.3389/fmed.2022.896409)
Supplement: Supplementary file 1 [file Data_Sheet_1.pdf]

Alterations in bile acids as metabolic signatures in the patients with human adenovirus type 7 infection

Wen Xu[1#], Juan Du[2,3#], Ting-Ting Wei[2,3#], Lin-Yi Chen[2,3], Xin-Xin Yang[1], Tu Bo[1], Han-Yu Liu[2,3], Ming-Zhu Xie[2,3], Tian-Shuo Zhao[2,3], Jun-Lian Yang[1], Fuqiang Cui[2,3*], Wei-Wei Chen[1*], Qing-Bin Lu[2,3*]

Materials and Methods

LC–MS analysis of samples

Reversed-phase analysis was performed on a Waters ACQUITY Ultra Performance LC system using an ACQUITY UPLC BEH C18 analytical column (i.d., 2.1 mm× 100 mm; particle size 1.7 mm; pore size, 130 Å). Water/formic acid (99.9:0.1 v/v) was used as mobile phase A and acetonitrile/formic acid (99.9:0.1 v/v) as mobile phase B. A linear gradient LC system (Waters, Milford MA) was optimized as follows: the composition of mobile phase B was changed from 3% to 80% in 7 min, reached 98% in 8 min and held for 5 min, and then reached 100% in 1 min and held for 3 min. The temperature of the sample manager was set at 4℃, with an injection volume of 2 ml for each analysis. The QC samples were injected at regular intervals (every 10 samples) throughout the analytical run. These inserted QC samples were used to evaluate the repeatability of sample pretreatment and monitor the stability of the LC-MS system during sequence analysis. A Waters Q-TOF Premier mass spectrometer was used to perform the mass spectrometry in positive ion electrospray mode. The instrumental parameters were set as follows: The mass scan range was 50 m/z-1000 m/z using an accumulation time of 0.2 s per spectrum; the MS acquisition rate was set to 0.3 s with a 0.02 s inter scan delay; high-purity nitrogen was used as nebulizer and drying gas. The nitrogen drying gas was at a constant flow rate of 600 L/h, and the source temperature was 120℃. For the positive mode, the capillary voltage was set at 3.0 kV and the sampling cone voltage was set at 45.0 V. Argon was used as collision gas. MS/MS analysis was performed on the mass spectrometer set at different collision energies of 10 eV-50 eV according to the stability of each metabolites. The time of flight analyzer was used in V mode and tuned for maximum resolution (>10,000 resolving power at m/z 556.2771). The instrument was previously calibrated with sodium formate; the lock mass spray for precise mass determination was set by leucine enkephalin at 556.2771 m/z with concentration of 0.5 ng/L in the positive ion mode. All analyses were acquired using the lock spray to ensure accuracy and reproducibility.

Detection of serum cytokine for the patients and asymptomatic cases

Serum concentrations of interleukin (IL) 6 (Abcam, USA, ab46027), interferon (IFN) γ (Abcam, USA, ab46048), interferon-inducible protein (IP) 10 (Abcam, USA, ab173194), interferon alpha 1 (Abcam,, USA, ab213479), IL-10 (Abcam USA, ab100549), HMGB-1 (IBL, Germany, ST51011), and serum receptor for advanced glycation end-product (sRAGE, R&D, USA, DRG00) were detected by commercial ELISA kits following instructions provided by manufacturer. Briefly, 50 μL serum samples and standards were added into plate coated with specific capture antibodies. After incubation, detection antibodies tagged with HRP were added. Following incubation for an appropriated time, unbounded antibodies were removed by several times washing and then TMB substrate solution was added. At last, the reaction was stopped by stop solution. The optical density (O.D.) of every well was measured at 450 nm by microplate reader. The concentrations of samples were calculated using standard curve.

Table S1. Basic information for the subjects in the study

Variable	Healthy controls (n=14)	HAdV-7 Patients (n=35)	HAdV-7 asymptomatic cases (n=32)	P value
Age, years, Mean±SD	21±1	20±2	21±1	0.075*
Sex, male, n (%)	14 (100)	35 (100)	32 (100)	1.000#
Body mass index, Kg/m^2, Mean±SD	22.1±1.0	23.1±4.8	21.7±1.1	0.223*
Smoking, n (%)	7 (50.0)	17 (47.0)	16 (50.0)	0.992#
Drinking, n (%)	2 (14.3)	10 (28.6)	5 (15.6)	0.342#
Diagnosis, n (%)				
Upper respiratory tract infection	-	21 (60.0)	-	
Pneumonia	-	14 (40.0)	-	
Clinical manifestation				
Pharyngalgia	-	32 (91.4)	-	
Fever	-	30 (85.7)		
Cough	-	28 (80.0)	-	
Diarrhea	-	16 (45.7)	-	
Expectoration	-	15 (42.9)	-	
Chill	-	8 (22.9)	-	
Lymphadenectasis	-	8 (22.9)	-	
Headache	-	7 (20.0)		
Nausea	-	3 (8.9)	-	
Vomit	-	2 (5.7)	-	

*compared by one-way analysis of variance;

#compared by chi-square test.

SD, standard deviation.

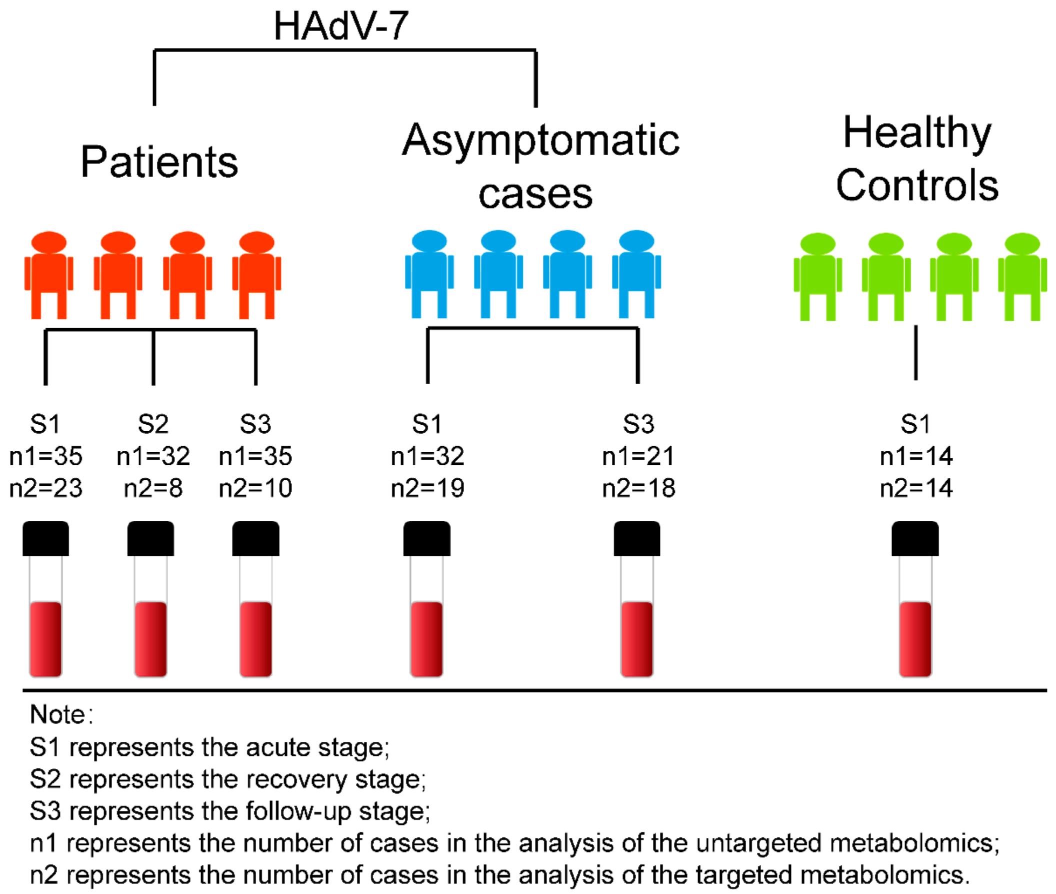

Fig S1. Flow charts of all the samples were collected and detected from patients, asymptomatic cases and healthy controls.

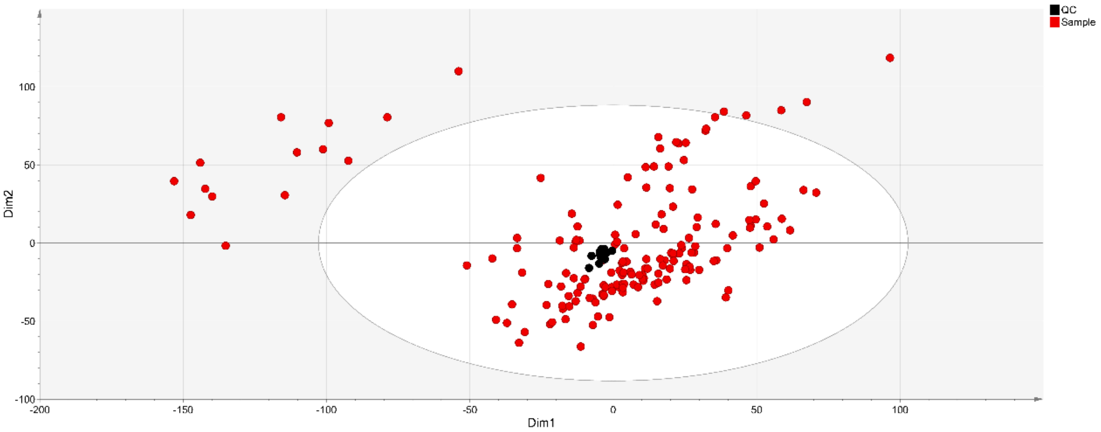

Fig S2. The PCA of all the samples, together with quality control samples.

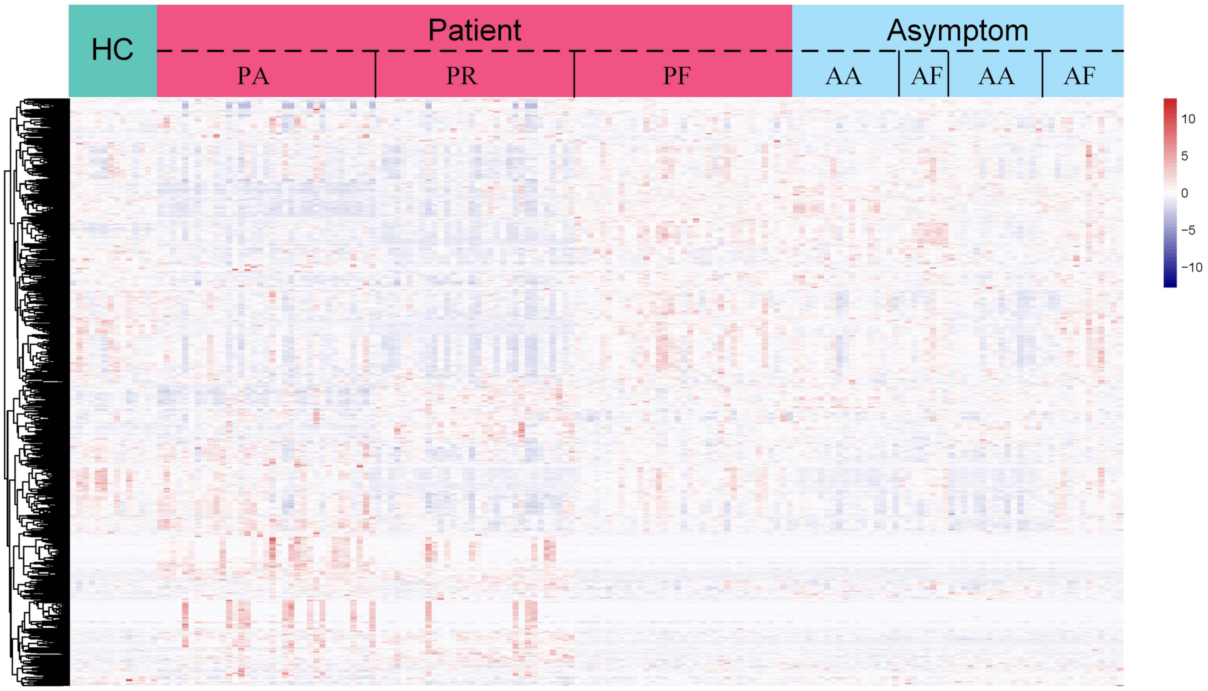

Fig S3. Heatmap of all the metabolites among patients, asymptomatic cases and healthy controls.

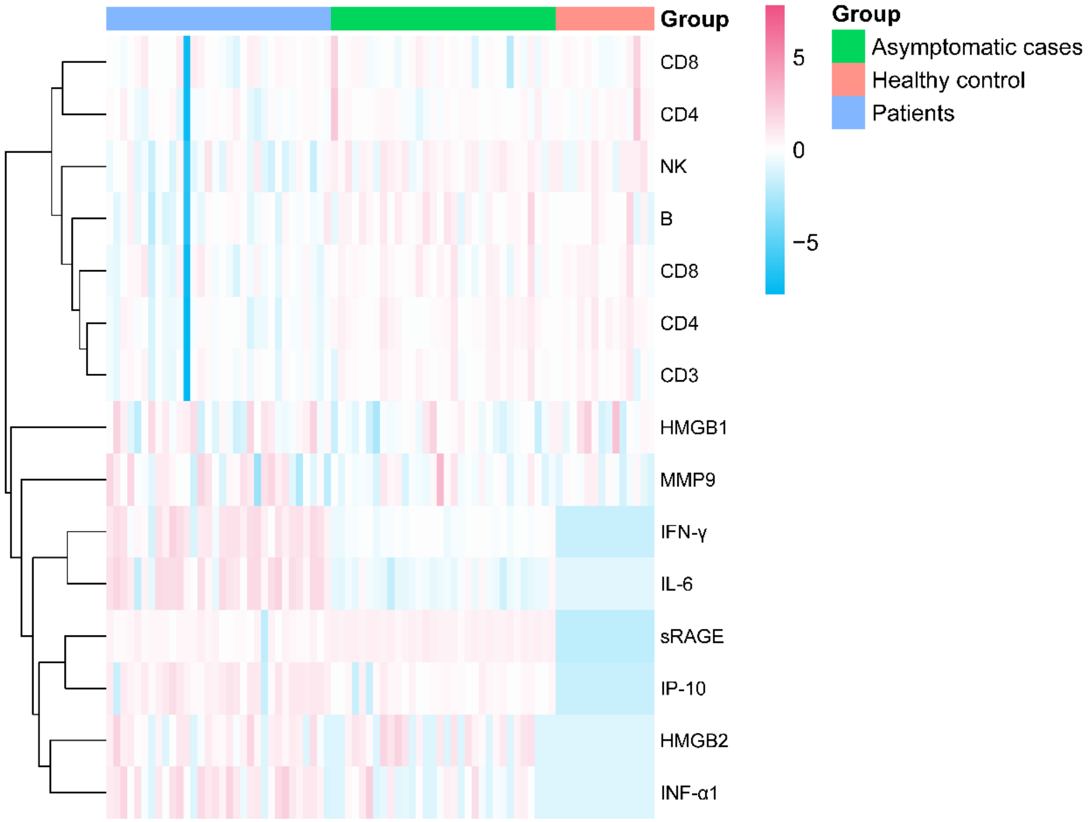

Fig.4. Heatmap of cytokines and peripheral lymphocyte subsets among patients, asymptomatic cases and healthy controls.
